# Invariance and item bias of the Mental Health Continuum Short-Form for South African university first-year students

**DOI:** 10.4102/ajopa.v6i0.143

**Published:** 2024-03-26

**Authors:** Karina Mostert, Leon de Beer, Ronalda de Beer

**Affiliations:** 1 Department of Management Cybernetics, Faculty of Economic and Management Sciences, North-West University, Potchefstroom, South Africa; 2 WorkWell Research Unit, Faculty of Economic and Management Sciences, North-West University, Potchefstroom, South Africa

**Keywords:** subjective well-being, Mental Health Continuum-Short Form, factorial validity, configural invariance, metric invariance, scalar invariance, item bias, internal consistency, first-year university students

## Abstract

Over the last decade, higher education institutions (HEIs) have become increasingly interested in student well-being. However, since the student population is very diverse in South Africa, questionnaires measuring the well-being of students must be psychometrically sound for different cultural and demographic groups. This study aimed to determine the psychometric properties of the Mental Health Continuum Short-Form (MHC-SF), including factorial validity, measurement invariance, item bias and internal consistency. The sample consisted of 1285 first-year university students. The three-factor structure of the MHC-SF was confirmed, indicating that emotional, social and psychological well-being are three independent factors. Invariance results showed that the MHC-SF produced similar results across campuses and gender sub-groups, although partial invariance was present among language groups. Item bias was present for different sub-groups, but the practical impact was negligible. Reliability scores indicated that all three dimensions are reliable in this sample. This study’s findings could help higher education institutions with preliminary results on the validity and reliability of a widely used well-being measure to assess university students’ subjective well-being and could aid in investigating and measuring first-year students’ overall well-being during their transition to tertiary education.

**Contribution:**

This study contributes to creating knowledge about fair and unbiased measurement of student well-being across different sub-groups in South Africa.

## Introduction

Student well-being is increasingly becoming a worldwide concern, also in South Africa (Jones et al., 2021; Pretorius & Blaauw, 2020). Many students, specifically first-year students, struggle with mental and psychological health problems such as anxiety (Wangeri et al., 2012), depression (Bakker et al., 2017), stress (Schwartz et al., 2021), panic attacks (Bruffaerts et al., 2019), eating disorders (Levine, 2012) and suicidal thoughts (Bruffaerts et al., 2019). There is also a growing concern as students experience more mental and psychological challenges (Browning et al., 2021). The *South Africa Journal of Psychology* published a special edition in 2021 to emphasise the importance of students’ well-being and mental health and to discuss the high suicide rates. Mental health issues may negatively influence many aspects of a student’s life, including their quality of life, academic success, future careers and earning potential (Eisenberg et al., 2007; Pascoe et al., 2020).

Well-being is critical to overcoming obstacles, achieving goals and living a happier life (Diener & Chan, 2011). Studies show that when students feel content, they concentrate better and retain knowledge more efficiently (Schneidermann et al., 2004). As a result, it could encourage students to be more involved in social contexts, allowing them to care about their own and others’ well-being and to assume leadership roles (Awartani et al., 2008; Schneidermann et al., 2004). Well-being is also related to retention and graduation rates in higher education (Schneidermann et al., 2004).

Corey Keyes developed the Mental Health Continuum-Short Form (MHC-SF) to measure mental health consisting of three factors: (1) emotional well-being (an individual’s life satisfaction, positive feelings, and quality of life), (2) social well-being (how well individuals function in their environment and the extent to which they feel they belong) and (3) psychological well-being (an individual’s capacity to grow and function independently) (Keyes, 2009).

Although this instrument is widely used to measure mental health (Joshanloo et al., 2013; Keyes, 2009; Westerhof & Keyes, 2010), it is vital that assessment has been scientifically proven to be accurate and reliable, equitable and unbiased for all individuals and groups. Since this is also important for student populations, this study aims to test the psychometric properties of the MHC-SF, specifically in a diverse group of first-year university students.

### Well-being as measured by the Mental Health Continuum-Short Form

According to Keyes (2002), hedonic well-being can be related to mental health. It includes components of subjective well-being and good functioning and depends on an individual’s perspective and appraisal of their livelihood and the quality of their existence (Keyes, 2002). Keyes (2002) continues by stating that emotional, social and psychological well-being are the three major components of subjective well-being. *Emotional well-being* is defined as the ability to develop pleasant emotions, moods, thoughts and sentiments and to adjust when faced with adversity and challenging conditions. Emotional well-being allows you to concentrate on the positive aspects of a situation while managing the unpleasant emotions and feelings that may arise (Keyes, 2002, 2014). *Psychological well-being*, on the other hand, refers to inter- and intrapersonal factors related to an individual’s functioning and evaluation (Keyes, 2014). Psychological well-being refers to how an individual relates to others and the self on an individual level and may include the following traits: self-acceptance, autonomy, personal development, healthy relationships, environmental mastery and a sense of purpose in life. Lastly, *social well-being* refers to social aspects such as consistency, upgrade, integration, acceptability and contribution (Keyes, 2002). Individuals with high levels of social well-being will embrace most aspects of society, regard themselves as contributing community members, and experience social well-being once they believe they are accepted by their environment (Keyes, 2002).

### Psychometric properties of the Mental Health Continuum-Short Form

*Factorial validity* is defined as ‘the extent to which items are designed to measure a particular factor (i.e., latent construct) and actually do so’ (Byrne, 2010, pp. 97–98). Concerning factorial validity, studies in Argentina (Lupano Perugini et al., 2017), Canada (Lamborn et al., 2018), the Netherlands (Kennes et al., 2020), South Korea (Lim, 2014) and France (Karaś et al., 2014) have all found the MHC-SF to be a valid and reliable measure. The three-factor structure (emotional, social and psychological well-being) is supported by numerous research studies testing the factorial validity of the MHC-SF in the adult population (Karaś et al., 2014; Kennes et al., 2020; Luijten et al., 2019; Lupano Perugini et al., 2017). A study conducted by Keyes et al. (2008) in South Africa confirmed the three-factor structure of the MHC-SF among Setswana-speaking people in South Africa, demonstrating that the three-factor structure observed in United States (US) samples may be replicated in South Africa (Keyes et al., 2008).

This study will consider three types of invariance. *Configural invariance* refers to the degree to which the factor structure can be accurately replicated across groups (i.e., the factor structure fits the data to the same degree and has the same pattern across all sub-groups). *Metric invariance* indicates if each item contributes equally to the latent construct across different sub-groups (i.e., items have equal factor loadings across sub-groups). *Scalar invariance* requires the test scores to have the same meaning and interpretation across sub-groups (He & Van de Vijver, 2012; Van de Vijver & Tanzer, 2004).

Concerning the invariance of the MHC-SF, a study conducted in the United States and Iran demonstrated partial metric invariance between the two countries and full metric invariance between gender groups (Joshanloo, 2016). In a cross-cultural study, including 38 countries and 8066 university students, Żemojtel-Piotrowska et al. (2018) found that configural invariance could not be confirmed across cultural groups included in the study because of the model’s complexity. In a sample of 624 respondents from various South African organisations, Van Zyl and Olckers (2019) established good metric invariance across gender groups but could not establish invariance for age cohorts, language groups (Afrikaans, English and African groups) or marital status.

Item bias occurs when participants across different groups interpret an item differently based on item-level incongruities and not necessarily because of actual differences in the underlying construct. Item bias occurs because of several reasons, including the appropriateness of the item content based on group specifics, connotative meaning attached to the item, poor item translation, ambiguous items and different response styles (He & Van de Vijver, 2012; Van de Vijver & Tanzer, 2004). Concerning the item bias of the MHC-SF, there have been concerns with item content and wording in previous validation studies, particularly with the social subscale (Santini et al., 2020). Previous studies conducted among 38 countries, including South Africa, indicated that emotional content is less biased and more universal among cultures (Żemojtel-Piotrowska et al., 2018), while psychological and social items show more cultural variation.

The MHC-SF showed high *internal consistency* and discriminant validity in a sample of adolescents and adults from the United States, the Netherlands and South Africa (Keyes, 2009). In a sample of adult Setswana speakers, the following Cronbach’s alpha coefficients were reported: emotional well-being, α = 0.70; psychology well-being, α = 0.67; and social well-being, α = 0.59; however, the overall internal consistency was acceptable with α = 0.74 (Keyes et al., 2008).

Van Zyl and Olckers (2019) showed the scale has Cronbach’s alpha coefficients of 0.86 for emotional well-being, 0.88 for social well-being and 0.86 for psychological well-being. Among student samples, it was found that the internal consistency of the subscales was satisfactory, with Cronbach’s alpha coefficients ranging from 0.79 to 0.85 for emotional well-being, 0.63 to 0.80 for social well-being, and 0.81 to 0.86 for psychological well-being (Foster & Chow, 2019; Joshanloo et al., 2017; Lamers et al., 2011).

### Study objectives

Although a range of psychometric properties could be considered when instruments are validated, this study focused on testing the factorial validity, structural invariance, metric invariance, scalar invariance, item bias and internal consistency of the MHC-SF.

## Methods

### Participants

The characteristics of the participants can be seen in Table 1.

**TABLE 1:** Characteristics of the participants.

Item	Category	Frequency	%
Age (in years)	17–20	672	52.2
	21–22	449	35.0
	23–24	164	12.8
Home language	Afrikaans	511	39.8
	English	102	7.9
	Sepedi	43	3.3
	Sesotho	114	8.9
	Setswana	339	26.4
	siSwati	18	1.4
	Tshivenda	12	0.9
	isiNdebele	5	0.4
	isiXhosa	41	3.2
	isiZulu	64	5.0
	isiTsonga	22	1.7
	Missing values	14	1.1
Campus	Campus 1	343	26.7
	Campus 2	713	55.5
	Campus 3	220	17.1
	Missing values	9	0.7
Gender	Male	410	31.9
	Female	866	67.4
	Missing values	9	0.7
Ethnicity	Asian people	3	0.2
	Black people	693	53.9
	Mixed race people	60	4.7
	Indian people	18	1.4
	White people	506	39.4
	Other people	5	0.4

Note: Where percentages do not sum to a 100, it is due to missing values.

The sample consisted of 1285 first-year university students studying at three different campuses at a South African university. In total, 672 (72.4%) were between the ages of 17 and 20 years, and 449 (35%) were between 21 and 22 years. Of these participants, 511 (39.8%) were Afrikaans, followed by 339 (26.4%) Setswana-speaking individuals, while 114 (8.9%) spoke Sesotho. Regarding gender, there were 410 (31.9%) male participants and 886 (67.4%) female participants. Most participants were black students (53.9%), followed by white students. The three campuses each has a unique and diverse culture. The first campus is considered peri-urban (i.e., an urban area adjacent to rural communities) and ideal for academic programmes focusing on rural development in indigenous knowledge. The second campus is the biggest and oldest of the three campuses and is situated in a town that is considered a suburban region. The third campus is the smallest and situated near a metropolitan city in an urban region. This campus hosts students from different cultures and language groups. Most of the sample was enrolled at campus 2 (55.5%), while campuses 1 and 3 make up for the other 45.5% of participants.

### Instrument

The MHC-SF comprises 14 items representing various aspects of well-being (Keyes, 2002). Each element represents a subjective sense of well-being rated according to the frequency over the last month on a Likert-type scale ranging from 1 (never) to 6 (every day). The instrument compromises three subscales: emotional well-being is measured by the first three items (e.g., ‘How frequently did you felt joyful’). Five items assess social well-being (i.e., ‘How frequently did you have a sense of belonging to a community?’). Six items measure psychological well-being (e.g., ‘How frequently did you feel at ease with the responsibilities of daily life?’).

### Procedure

A secure link was placed on the course module portals for courses first-year students from various disciplines across campuses were enrolled for. Throughout the study’s duration, students were encouraged to contribute voluntarily. Trained field workers presented brief awareness sessions in the corresponding classrooms. Pertinent information about the study’s goal and objectives was provided during these sessions. Completion took around 15 min – 20 min.

### Data analysis

Both measurement invariance testing and Differential Item Functioning (DIF) analysis were implemented to ensure the scales were accurate and unbiased across different groups. Differential Item Functioning analysis enables specific items in scales to be identified that might be interpreted differently by different groups, measurement invariance testing ensured that the overall construct measured remained consistent across these groups. Together, these methods provided a comprehensive approach.

The statistical modelling program Mplus 8.6 (Muthén & Muthén, 2021) was used to examine the psychometric properties of the scale. Factorial validity was determined using confirmatory factor analysis (CFA) (Brown, 2015). Maximum likelihood estimation was used, with the covariance matrix as input (Muthén & Muthén, 2021). Based on the findings of previous validation studies reported in the literature, a three-dimensional structure was tested (emotional, social and psychological well-being), compared with an additional one-factor model. The following fit indices were used to evaluate the fit of the metric models: the *χ*² statistic, the comparative fit index (CFI), the Tucker-Lewis index (TLI), the root mean square error of approximation (RMSEA) and the standardised root mean square residual (SRMR).

A suitable fit is a CFI and TLI value of 0.90 or higher, a RMSEA and a SRMR value of less than 0.08 (Byrne, 2001; Hu & Bentler, 1999; Ed. Hoyle, 1995; Van De Schoot et al., 2012). The Akaike information criterion (AIC) and sample-adjusted Bayesian information criterion (BIC) were used to compare the fit of competing models – lower values indicate better fit. It should be noted that these cut-off points should only be regarded as guidelines, as there is little agreement on the values for good fit (Lance et al., 2016).

Measurement invariance was also investigated based on language, campus and gender groups. Multigroup analysis was used that included three models:

- The *configural invariance* model tests if the factor structure is analogous across sub-groups and is the baseline model for the more constrained models.
- The *metric invariance* model assumes similarity or invariance of the factor loading across different sub-groups.
- The *scalar invariance* model tests if the factor loadings and item intercepts are similar or invariant across different sub-groups (Preti et al., 2013).

Comparative fit index and RMSEA were used as cut-off points when testing for metric invariance. In addition, changes in CFI were used (Shi et al., 2019). A difference in the CFI of less than 0.01 between two nested models indicates that the model’s fit does not deteriorate significantly (Choi et al., 2011; Rudnev et al., 2018). If the more constrained model is rejected, loadings of items are freed to establish partial metric invariance (Cheung & Rensvold, 2002; Preti et al., 2013). At least two factor loadings and intercepts must remain specified to be equal across groups for partial measurement invariance to hold (see Van de Schoot et al., 2013).

Differential item functioning was used to test for the presence of item bias for (1) the four official language groups of the participating university (Afrikaans, Setswana, Sesotho and English); (2) for three different campuses of the university; (3) and gender sub-groups. The *lordif* package (Choi et al., 2011) in *R*Studio (https://www.rstudio.com/) was used to test the presence of uniform and non-uniform item bias. Uniform bias occurs when groups are compared and systematic differences are detected in the underlying construct, while non-uniform bias occurs when there is a likelihood that answers on an item will differ or fluctuate across sub-groups (Teresi & Fleishman, 2007).

Ordinal logistic regression was used to generate three likelihood-ratio *χ*² statistics using the following formulas to compare models to determine uniform and/or non-uniform bias (Choi et al., 2011):

$$\text{Model }0\;(\text{Baseline}):\text{logit }P\;(u_{i}\geq k)=\alpha_{k}$$ [Eqn 1]

$$\text{Model }1\;(\text{Ability}):\text{logit }P\;(u_{i}\geq k)=\alpha_{k}+\beta_{1}*\text{ability}$$ [Eqn 2]

$$\text{Model }2\;(\text{Ability and Group}):\text{logit }P\;(u_{i}\geq k)=\alpha_{k}+\beta_{1}*\text{ability}+\beta_{2}*\text{group}$$ [Eqn 3]

$$\text{Model }3\;(\text{Interaction}):\text{logit }P\;(u_{i}\geq k)=\alpha_{k}+\beta_{1}*\text{ability}+\beta_{2}*\text{group}+\beta_{2}*\text{ability}*\text{group}$$ [Eqn 4]

Using DIF, biased items can be detected when the log-likelihood values of models are compared and statistically significant differences are detected (*p* < 0.01). Because the *lordif* package (Choi et al., 2011) in *R*Studio was used for the analyses in this study, only dimensions with more than three items could be tested. Therefore, the results of item bias were analysed for the emotional and psychological well-being items. Uniform bias is seen when comparing logistic Models 1 and 2 ($\chi_{12}^{2}$; *df* = 1). Non-uniform bias is detected when comparing logistic Models 2 and 3 ($\chi_{23}^{2}$; *df* = 1). Total bias is detected when comparing logistic Models 1 and 3 ($\chi_{13}^{2}$; *df* = 2) (Choi et al., 2011). In summary, comparing Model 1 (which only considers ability) with Model 2 (which adds group information), uniform bias was assessed – that is, whether a group’s responses are consistently different, regardless of their ability level. Then, by comparing Model 2 with Model 3 (which also considers the interaction between ability and group), non-uniform bias, where the difference in responses might change at different ability levels, was assessed. Finally, by comparing Model 1 directly with Model 3, total bias, combining both uniform and non-uniform biases, to see the overall impact of group membership on responses is assessed.

The pseudo-McFadden *R*^2^ statistic was used to test the impact or practically significant effect of the DIF. The magnitude of DIF can be classified as negligible (< 0.13), moderate (between 0.13 and 0.26), and large (> 0.26) (Zumbo, 1999). In addition, the impact of uniform DIF can be determined using the difference in the *β*_1_ coefficient from Models 1 and 2. The practical significance of uniform DIF was determined with 10% differences between Models 1 and 2, which indicates a practically meaningful effect (Crane et al., 2004). Lower 5% and even 1% thresholds are also used (Crane et al., 2007).

Cronbach’s alpha coefficient was used to determine the reliability of the scales, with α ≥ 0.70 indicating internal consistency (Nunnally & Bernstein, 1994). In addition, McDonald’s omega was calculated and reported for a more accurate estimation of internal consistency (Cortina et al., 2020). Reliability coefficients ≥ 0.70 indicate good internal consistency (Kline, 2015).

### Ethical considerations

The study was approved by the Ethics Committee-Faculty of Economic and Management Sciences (EC-EMS) (ethics number: NWU-HS-2014-0165-A4). Participants signed an informed consent form before completing the questionnaire. Participants completed the questionnaires anonymously and were assured that their reported responses would adhere to the project’s confidentiality criteria and that the findings would be stored in a secure database that is password protected.

## Results

### Factorial validity

The hypothesised three-factor structure of the MHC-SF was a good fit for the data (*χ*^2^ = 462.36 (*p* < 0.001); *df* = 74; CFI = 0.94; TLI = 0.93; RMSEA = 0.07; SRMR = 0.04; AIC = 48570.78; BIC = 48797.13) when compared to the one-factor model (*χ*^2^ = 1262.86; *df* = 78; CFI = 0.82; TLI = 0.79; RMSEA = 0.12; SRMR = 0.08; AIC = 50478.66; BIC = 50684.89). Therefore, further analyses were based on the three-factor model. Table 2 indicates the results for the items’ standardised loadings for the MHC-SF latent variables. The factor loadings were all rule of thumb values of 0.50 and even 0.70 (Hair et al., 2015).

**TABLE 2:** Standardised factor loadings.

Factors	Item	Loading	s.e.	*p*
Emotional well-being	1	0.78	0.019	0.000
	2	0.89	0.013	0.000
	3	0.87	0.013	0.000
Social well-being	4	0.71	0.022	0.001
	5	0.78	0.017	0.000
	6	0.87	0.012	0.000
	7	0.75	0.020	0.000
	8	0.75	0.019	0.000
Psychological well-being	9	0.73	0.020	0.000
	10	0.73	0.019	0.000
	11	0.72	0.020	0.000
	12	0.72	0.021	0.000
	13	0.76	0.020	0.000
	14	0.83	0.012	0.000

Note: All *p*-values < 0.001.

s.e., standard error.

The correlation matrix for the latent variables is presented in Table 3.

**TABLE 3:** Estimated correlation matrix.

Variables	1	2	3
1. Emotional well-being	1.00	-	-
2. Social well-being	0.70	1.00	-
3. Psychological well-being	0.79	0.76	1.00

The results in Table 2 show that emotional, social and psychological well-being are correlated. All relationships had a large effect size (*r* ≥ 0.50; Sullivan & Feinn, 2012).

#### Measurement invariance

Next, the measurement invariance of the MHC-SF was examined. As previously stated, most measurement invariance tests include configural, metric and scalar invariance testing. The results of measurement invariance across language, campus and gender groups are shown in Table 4.

**TABLE 4:** Summary of measurement invariance analyses for the Mental Health Continuum Short-Form.

Language	*χ* ^2^	*df*	CFI	ΔCFI	RMSEA	ΔRMSEA
**Configural**	716.43	296	0.940	-	0.073	-
Metric	776.27	329	0.936	−0.004	0.071	−0.002
Scalar	901.77	362	0.923	−0.013	0.075	0.004
Partial Scalar	879.69	361	0.926	−0.010	0.073	−0.002
**Campus**						
Configural	641.24	222	0.940	-	0.071	-
Metric	674.87	244	0.938	−0.002	0.069	−0.002
Scalar	760.88	266	0.929	−0.009	0.071	0.002
**Gender**						
Configural	615.98	148	0.935	-	0.070	-
Metric	637.19	159	0.933	−0.002	0.069	−0.001
Scalar	672.61	170	0.930	−0.003	0.068	−0.001

*χ*^2^, Chi-square; *df*, degrees of freedom; CFI, comparative fit index; ΔCFI, delta (change in) CFI; RMSEA, root mean square error of approximation; ΔRMSEA, delta (change in) RMSEA.

As can be seen from Table 4, gender and campus did not show any deviation above 0.01 for CFI and can, therefore, be considered invariant across those groups. Delta changes in CFI were all below or on the cut-off of 0.01, except for the scalar model of the language groups. The four language groups used in the analyses were Afrikaans, English, Sesotho and Setswana. However, partial scalar invariance was reached by freeing the intercept of Item 12 in the Afrikaans group, indicating that means could still be compared for different language groups if necessary.

### Item bias

Even though the invariance results are encouraging, bias was also tested across sub-groups of language, campus, and gender to test for uniform, non-uniform and total bias.

#### Differential item functioning based on language

As can be seen from Table 5, no bias was detected for emotional well-being. However, uniform and total bias were detected for Item 2 for social well-being and Items 1 and 3 for psychological well-being (*p* < 0.01 for $\chi_{12}^{2}$ *u* and $\chi_{13}^{2}$), although the practical impact can be considered negligible, as seen from the pseudo-McFadden *R*^2^ values smaller than 0.13 and Δ*β*1 coefficients smaller than 0.05 (5%).

**TABLE 5:** Differential item functioning for language.

Group	Item	$\chi_{12}^{2}$ *u*	$\chi_{13}^{2}$ t	$\chi_{23}^{2}$ n	Δ*β*1	$R_{12}^{2}$	$R_{13}^{2}$	$R_{23}^{2}$
Social	Item 1	0.6927	0.2960	0.1206	0.0029	0.0004	0.0019	0.0015
	Item 2	**0.0001**	**0.0004**	0.2248	0.0225	0.0054	0.0065	0.0012
	Item 3	0.6244	0.4211	0.2345	0.0036	0.0005	0.0016	0.0011
	Item 4	0.9666	0.6645	0.2809	0.0004	0.0001	0.0011	0.0010
	Item 5	0.7253	0.8128	0.6476	0.0025	0.0003	0.0008	0.0004
Psychological	Item 1	**0.0003**	**0.0000**	0.0052	0.0036	0.0058	0.0097	0.0039
	Item 2	0.0820	0.0482	0.1123	0.0047	0.0019	0.0036	0.0017
	Item 3	**0.0000**	**0.0000**	0.0276	0.0568	0.0147	0.0172	0.0025
	Item 4	0.1854	0.5485	0.9862	0.0028	0.0014	0.0015	0.0000
	Item 5	0.0409	0.0582	0.2708	0.0126	0.0023	0.0034	0.0011
	Item 6	0.0818	0.0528	0.1252	0.0014	0.0019	0.0035	0.0016

Note: Values are indicated in bold text when the log-likelihood values of models are compared and statistically significant differences are detected (*p* < 0.01).

$\chi_{12}^{2}$, Chi-square of model 1 compared to model 2; $\chi_{13}^{2}$, Chi-square of model 1 compared to model 3; $\chi_{23}^{2}$, Chi-square of model 2 compared to model 3; *β*_1_, change in beta coefficient; $R_{12}^{2}$, pseudo-Mcfadden *R*^2^ of model 1 compared to model 2; $R_{13}^{2}$, pseudo-Mcfadden *R*^2^ of model 1 compared to model 3; $R_{23}^{2}$, pseudo-Mcfadden *R*^2^ of model 2 compared to model 3.

*u*, uniform bias; *t*, total bias; *n*, non-uniform bias.

#### Differential item functioning based on campus

The results of the differential item functioning based on campus can be seen in Table 6. The model comparisons showed significant differences for three of the psychological items (items 1, 3 and 6), indicating uniform and total bias for these items (*p* < 0.01 for $\chi_{12}^{2}$ *u* and $\chi_{13}^{2}$). These effects were also negligible, based on the pseudo-McFadden *R*^2^ values smaller than 0.13 and Δ*β*1 coefficients smaller than 0.05 (5%).

**TABLE 6:** Differential item functioning for campus.

Group	Item	$\chi_{12}^{2}$ *u*	$\chi_{13}^{2}$ t	$\chi_{23}^{2}$ n	Δ*β*1	$R_{12}^{2}$	$R_{13}^{2}$	$R_{23}^{2}$
Social	Item 1	0.2135	0.1753	0.1969	0.0011	0.0008	0.0016	0.0008
	Item 2	0.3230	0.1254	0.0843	0.0033	0.0006	0.0018	0.0012
	Item 3	0.0616	0.1577	0.5942	0.0080	0.0014	0.0017	0.0003
	Item 4	0.4317	0.6246	0.6274	0.0006	0.0004	0.0007	0.0002
	Item 5	0.1352	0.2464	0.4909	0.0023	0.0010	0.0014	0.0004
Psychological	Item 1	**0.0002**	**0.0001**	0.0631	0.0027	0.0050	0.0066	0.0016
	Item 2	0.0397	0.0680	0.3195	0.0027	0.0017	0.0023	0.0006
	Item 3	**0.0000**	**0.0000**	0.0396	0.0319	0.0067	0.0084	0.0017
	Item 4	0.1476	0.3594	0.7657	0.0019	0.0011	0.0012	0.0001
	Item 5	0.4779	0.3984	0.2753	0.0036	0.0004	0.0011	0.0007
	Item 6	**0.0000**	**0.0000**	0.2862	0.0041	0.0061	0.0068	0.0007

Note: Values are indicated in bold text when the log-likelihood values of models are compared and statistically significant differences are detected (*p* < 0.01).

$\chi_{12}^{2}$, Chi-square of model 1 compared to model 2; $\chi_{13}^{2}$, Chi-square of model 1 compared to model 3; $\chi_{23}^{2}$, Chi-square of model 2 compared to model 3; *β*_1_, change in beta coefficient; $R_{12}^{2}$, pseudo-Mcfadden *R*^2^ of model 1 compared to model 2; $R_{13}^{2}$, pseudo-Mcfadden *R*^2^ of model 1 compared to model 3; $R_{23}^{2}$, pseudo-Mcfadden *R*^2^ of model 2 compared to model 3.

*u*, uniform bias; *t*, total bias; *n*, non-uniform bias.

#### Differential item functioning based on gender

Regarding the results of the differential item functioning based on gender, Table 7 shows no biased items for emotional and social well-being (*p* < 0.01 for $\chi_{12}^{2}$ *u* and $\chi_{13}^{2}$). Again, the pseudo-McFadden *R*^2^ values smaller than 0.13 and Δ*β*1 coefficients smaller than 0.05 (5%) show that the magnitude of this bias is not practically significant and, therefore, negligible.

**TABLE 7:** Differential item functioning for gender.

Group	Item	$\chi_{12}^{2}$ *u*	$\chi_{13}^{2}$ t	$\chi_{23}^{2}$ n	Δ*β*1	$R_{12}^{2}$	$R_{13}^{2}$	$R_{23}^{2}$
Social	Item 1	0.0930	0.1811	0.4405	0.0017	0.0007	0.0009	0.0002
	Item 2	**0.0004**	**0.0009**	0.2002	0.0017	0.0031	0.0035	0.0004
	Item 3	0.5702	0.7266	0.5737	0.0008	0.0001	0.0002	0.0001
	Item 4	0.8662	0.6865	0.3948	0.0003	0.0000	0.0002	0.0002
	Item 5	0.9589	0.7701	0.4709	0.0001	0.0000	0.0001	0.0001
Psychological	Item 1	0.3307	0.5639	0.6550	0.0006	0.0003	0.0003	0.0001
	Item 2	0.9084	0.8966	0.6506	0.0001	0.0000	0.0001	0.0001
	Item 3	0.1021	0.0935	0.1505	0.0038	0.0007	0.0012	0.0005
	Item 4	0.0226	0.0367	0.2345	0.0063	0.0015	0.0019	0.0004
	Item 5	0.0918	0.1441	0.3099	0.0002	0.0008	0.0010	0.0003
	Item 6	0.4026	0.1165	0.0578	0.0001	0.0002	0.0012	0.0001

Note: Values are indicated in bold text when the log-likelihood values of models are compared and statistically significant differences are detected (*p* < 0.01).

$\chi_{12}^{2}$, Chi-square of model 1 compared to model 2; $\chi_{13}^{2}$, Chi-square of model 1 compared to model 3; $\chi_{23}^{2}$, Chi-square of model 2 compared to model 3; *β*_1_, change in beta coefficient; $R_{12}^{2}$, pseudo-Mcfadden *R*^2^ of model 1 compared to model 2; $R_{13}^{2}$, pseudo-Mcfadden *R*^2^ of model 1 compared to model 3; $R_{23}^{2}$, pseudo-Mcfadden *R*^2^ of model 2 compared to model 3.

*u*, uniform bias; *t*, total bias; *n*, non-uniform bias.

### Internal consistency

According to Nunnally and Bernstein (1994), Cronbach’s alpha reliability is acceptable when α ≥ 0.70. All factors had an acceptable coefficient: emotional well-being (α = 0.88), social well-being (α = 0.87) and psychological well-being (α = 0.89). In addition, McDonald’s omega (ω) was 0.88 for emotional well-being, 0.88 for social well-being and 0.89 for psychological well-being, indicating good internal consistency (Kline, 2015).

## Discussion

This study aimed to test the psychometric properties of the MHC-SF to determine if this instrument is valid and reliable for assessing first-year university students’ subjective well-being. The study’s primary objective was to determine the factorial validity, metric, scalar and structural invariance, item bias and internal consistency.

Confirmatory factor analysis was used to test the *factorial validity* of the MHC-SF. Two measurement models were tested, a one-factor and a three-factor model. The three-factor model showed a very good fit based on the fit indices. These findings are in line with prior validation studies conducted on the MHC-SF in Argentina (Lupano Perugini et al., 2017), Canada (Lamborn et al., 2018), the Netherlands (Kennes et al., 2020), South Korea (Lim, 2014) and France (Karaś et al., 2014). Therefore, this study confirms that mental health, as measured by the MHC-SF, consists of three distinct factors – emotional, social and psychological well-being.

*Measurement invariance* is viewed as a prerequisite for any study involving cross-cultural evaluation (He & Van de Vijver, 2012), emphasising the level of measurement at which scores from different sub-groups are compared (Van de Vijver & Tanzer, 2004). The configural, metric and scalar models were compared to test for invariance across language, campus and gender groups. Configural, metric and scalar invariance were confirmed for all groups included, although the intercept of Item 12 in the Afrikaans group had to be freed to reach partial scalar invariance for language, indicating that means could still be compared based on the language if required. This study’s results are slightly different from the results of Joshanloo (2016) and Van Zyl and Olckers (2019).

*Item bias* was tested using DIF. No bias was found for the three items measuring emotional well-being. Item 2 of social well-being showed uniform and non-uniform bias for language and gender sub-groups, while psychological well-being Items 1 and 3 showed uniform and non-uniform bias for language and campus sub-groups and Item 6 for campus sub-groups.

However, based on the pseudo-McFadden *R*^2^ values smaller than 0.13 and Δ*β*1 coefficients smaller than 5%, it can be concluded that, although statistically significant bias was detected, these values were not of practical magnitude or impact. These findings are in line with the findings of Żemojtel-Piotrowska et al. (2018) and Santini et al. (2020) and confirm that the content of the emotional well-being items is less biased, while social and psychological items can be more complicated to interpret between different sub-groups (in this case, language, campus and gender).

The Cronbach’s alpha coefficient was calculated to determine the internal consistency of the MHC-SF and found acceptable internal consistencies: emotional well-being (α = 0.88), social well-being (α = 0.87) and psychological well-being (α = 0.89). The internal consistency of the three subscales has previously been found to be adequate in student samples, with Cronbach’s alpha coefficients ranging from 0.79 to 0.85 for emotional well-being, 0.81 to 0.86 for psychological well-being, and 0.63 to 0.80 for social well-being (Foster & Chow, 2019; Joshanloo et al., 2017; Lamers et al., 2011).

### Limitations and recommendations

The study’s primary focus was on first-year university students. To further evaluate the findings of this study, future research could include students from different academic year groups (pre-graduate and post-graduate). Most participants were either Afrikaans or Setswana-speaking and mainly female, which restricts a holistic view of all language and gender groups. Future studies can present an equal representation of the population demographics to address this limitation. The study was conducted at a specific South African university and should be replicated at other universities with their unique characteristics, campuses and demographics. Some items of the MHC-SF were biased, which could hinder the comparability of findings across groups in other studies (He & Van de Vijver, 2012). Although these effects were negligible (*p* < 0.01; Choi et al., 2011), more research is needed to determine if the bias found in this sample is also problematic in other student samples. In addition, partial scalar invariance was established because the intercept of Item 12 in the Afrikaans language group had to be released. Future studies should examine whether this item is problematic in other language groups. Thus, more research is needed to validate invariance across different student sub-groups.

## Conclusion

In conclusion, the MHC-SF seems to be a valid, reliable, invariant and unbiased instrument to measure first-year university students’ social, emotional and psychological well-being in this study. The results of this study can help this university apply the MHC-SF with the current knowledge about its psychometric properties in their unique context, especially among students dealing with high demands and limited resources when it comes to their studies and find possible solutions to assist them. Valid and reliable measurement can also help students develop better awareness and knowledge of their subjective well-being.
